# Evaluating Antimycobacterial Screening Schemes Using Chemical Global Positioning System-Natural Product Analysis

**DOI:** 10.3390/molecules25040945

**Published:** 2020-02-20

**Authors:** Muaaz Mutaz Alajlani, Anders Backlund

**Affiliations:** 1 Pharmacognosy Research Group, Department of Medicinal Chemistry—Faculty of Pharmacy, Uppsala University, BMC—Biomedical Center, Box 574, S-751 23 Uppsala, Sweden; anders.backlund@ilk.uu.se; 2 Department of Pharmaceutical Biology/Pharmacognosy, Institute of Pharmacy, University of Halle-Wittenberg, Hoher Weg 8, DE 06120 Halle (Saale), Germany

**Keywords:** evaluation, screening, chemography, chemical property space, antimycobacteria, ChemGPS-NP, data evaluation, tuberculosis

## Abstract

Most of the targeted discoveries in tuberculosis research have covered previously explored chemical structures but neglected physiochemical properties. Until now, no efficient prediction tools have been developed to discriminate the novelty of screened compounds at early stages. To overcome this deficit, a drastic novel approach must include physicochemical properties filters provided by Chemical Global Positioning System-Natural Product analysis (ChemGPS-NP). Three different screening schemes GSK, GVKBio, and NIAID provided 776, 2880, and 3779 compounds respectively and were evaluated based on their physicochemical properties and thereby proposed as deduction examples. Charting the physiochemical property spaces of these sets identified the merits and demerits of each screening scheme by simply observing the distribution over the chemical property space. We found that GSK screening set was confined to a certain space, losing potentially active compounds when compared with an in-house constructed 459 highly active compounds (active set), while the GVKBio and NIAID screening schemes were evenly distributed through space. The latter two sets had the advantage, as they have covered a larger space and presented compounds with additional variety of properties and activities. The in-house active set was cross-validated with MycPermCheck and SmartsFilter to be able to identify priority compounds. The model demonstrated undiscovered spaces when matched with Maybridge drug-like space, providing further potential targets. These undiscovered spaces should be considered in any future investigations. We have included the most active compounds along with permeability and toxicity filters as supplemented material.

## 1. Introduction

*Mycobacterium tuberculosis*, the causative agent of tuberculosis (TB), infects one-third of the world population with traces back in history [1]. Today, TB still represents one of the most challenging threats to global human health, with over 2 billion people harboring latent infection and more than 9 million new cases reported every year. More than half a million of these cases eventually develop multi-drug-resistant (MDR) TB, and in recent years, extensively drug-resistant TB (XDR-TB) has been more frequently reported [2]. Nearly 2 million deaths are estimated to occur yearly [3]. Despite continuous and massive screening for potential antituberculosis agents, no breakthrough has been reported for over 40 years. Only 22 approved drugs [4] are available, out of which four drugs are considered first-line therapy [5]. Most of these first-line drugs in TB treatment were discovered during the 1950s and the 1960s, and only four drugs are today at phase three clinical trial. None are at phase one [6]. 

In late 2012, bedaquiline became the first novel TB drug approved in 40 years [7], due to the complexities that govern tuberculosis research. Bedaquiline itself is an orphan drug, and accelerated approval of the drug was sought, a type of temporary approval in the case of diseases lacking other viable treatment options. The challenge of meeting the expectations of this desired target product profile complicates drug discovery efforts. Considering how few drugs from the discovery stage successfully enter the TB clinical pipeline, an increased understanding of drug discovery hurdles should facilitate the development of novel intervention strategies [8]. Pharmaceutical companies are in constant search for new antituberculosis drugs with more favorable characteristics in terms of treatment period, toxicity reduction, targeting of MDR and even XDR, and possibly the ability to be coadministered with HIV medications. 

Dobson has defined chemical space to consist of compounds that are characterized by a wide range of chemical descriptors [9]. From this perspective, chemical space is defined to be the total descriptor space that encompasses all the small carbon-based molecules that could in principle be created [10]. Such a definition provides a huge advantage over any existing library, as roughly more than 99.9% of all small molecules that can be imagined have not been prepared and tested [11]. Despite the urgency of tuberculosis, a total of 42 compounds are the only drugs present for treatment. Information includes mechanisms and activity profiles on all approved drugs used to treat tuberculosis, on drugs in clinical development for TB, and on some already approved drugs being investigated for potential use in TB [12,13]. 

In this paper, we aim to chart and investigate different screening schemes of antituberculosis and provide insights on their distribution over the physicochemical space. We have characterized the overall chemical property space of antituberculosis compounds provided by ChemGPS-NP to navigate, validate, and predict antituberculosis compounds by providing a model for comparison.

## 2. Results and Discussion

There are continuous attempts by several laboratories to search for global antituberculosis compounds and subfragments, but none has reported a breach using virtual screening. Most of the success was achieved from locally active subsets with drastic measurements; in other words, the database library must fit the purpose of the experiment before its selection for screening, and this is usually done either by ligand-based or structural-based screening. These strategies consist of quantitative structure–activity relations (QSAR), 3D-QSAR, and pharmacophores. The approach of the hit-and-trail method has limited the screening strategies to their known chemical moieties and with an extremely poor hit rate [14,15].

Chemical property space has no such disadvantages, as similarity or dissimilarity is based on physicochemical properties rather than topology of chemical structure alone. ChemGPS-NP mapping can be a key factor in shaping such space as it accounts for over 50% of data variance. It reflects 35 major properties and characters, and the results are produced as principle components values.

**Figure 1:**
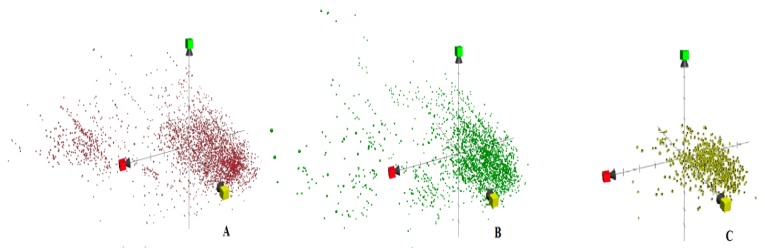
Different patterns revealed on plotting of (**A**) NIAID, (**B**) GVKBio, and (**C**) GSK on PC1 (x = red), PC2 (y = yellow) and PC3 (green) from ChemGPS-NP.

The chemical property space was charted by using the first three-dimensional values obtained from ChemGPS-NP for each compound. The first dimension (principal component one, PC1) represents size, shape, and polarizability; PC2 corresponds to aromatic and conjugation-related properties, while PC3 describes lipophilicity, polarity, and H-bond capacity. In that regard, each compound has a designated position in the chemical property space. Antituberculosis compounds from different data sets are scattered throughout the chemical property space, indicating a high diversity in form of physicochemical properties. This visualization method has constructed an easy way for data evaluation. The diversity in such properties is directly proportional to a higher discovery rate with potentially exclusive activities. In order to have more deducible indications, we have explored three screening schemes (GSK, GVKBio, and NIAID) from different suppliers using ChemGPS-NP. A great deal of variance was observed in accordance with the chemical property space of these sets but at the same time a common volume amongst these three schemes (Negative PC1, Positive PC2, and Positive PC3) (Figure 1). The common volume was noted by a higher density in their chemical property space. The screening scheme that lacked distribution in the chemical property space will have the disadvantage of missing potential targets, and the assays used should be investigated to ensure removing any limitation in the procedures.

**Figure 2:**
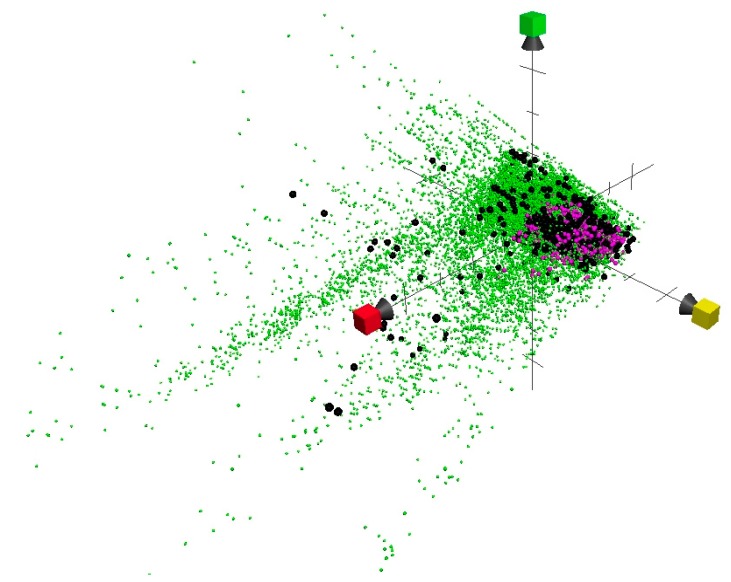
Chemical property space revealed as 3D Graphing (red axis = PC1, corresponding to size parameters; yellow axis = PC2, corresponding to aromaticity and conjugation related parameters; and green axis = PC 3, corresponding mainly to lipophilicity), compounds are contrasted by three colored sets; black representing antituberculosis compounds with highest activity, pink color represents GSK screening set, and green set represents the Maybridge screening collection. From this figure, it is obvious that the GSK did not cover potential targets presented by the active set (black spheres).

Obviously, variation is required to attain novel antituberculosis agents, but at the same time, we have observed that the GSK screening set was more confined to the common volume. This would indicate a safer or communal approach used by the company or a selective screening assay, especially that many active compounds were observed out in that volume. The proposed antimycobacterial chemical property space identified such merits and demerits alongside screening results and more usefully detected such problems and direct screening procedures towards their targets. High-throughput screening (HTS) and even Ultra HTS (uHTS) have become standard methods in the drug discovery [16]; unfortunately, they are still expensive techniques, as they require a library of semi-pure or preferably pure compounds at sufficient quantities, as well as more reliable assays that consider cost and validities. There are many compounds that have come from really expensive and time-consuming screen protocols, but they have produced very limited results, and no breakthrough was ever recorded in the field of tuberculosis research. Interestingly, this visualizing model presented in the chemical property space can indicate potential similarity that could be easily singled out from the proposed library, making screening enrichment a straightforward approach. The demerits in the GSK scheme were further demonstrated by incorporating it into the active set (459 compounds) and Maybridge screening collection (31,236 compounds) (Figure 2). Plotting these three sets together demonstrates the disadvantages of the confined GSK volume compared to NIAID and GVKBio schemes. As many highly active compounds (active set) were out of the range and far from GSK compounds. The 776 antituberculosis compounds of GSK have been proposed from GSK 2 million compounds yet missed potential active volumes in the highly active antituberculosis physicochemical space. The GSK example showed the importance of incorporating the physicochemical studies in screening programs, therefore, to cover all potential targets and to correct such deficiency at earlier stages.

The concept of using chemical properties in virtual screening and data mining is becoming clearer to scientists. An extra dimension would not only enrich hit rate percentages but would also aid in distinguishing activities and contribute to the understanding of mechanisms of action by including the chemical properties [17]. Compounds tagged with the highest antituberculosis activity (black spheres) as plotted, along with Maybridge compound library, can be the bases of further selection. In this regard, we propose the 8-dimensional Euclidian distance calculation that further determines selection. Compounds with antituberculosis activity could be sorted according to Euclidean distances to determine the nearest highly active compound. The Maybridge screening collection (http://www.maybridge.com) is generally considered as consisting of drug-like compounds. These compounds have favorable drug properties and therefore may constitute a supreme database for screening. Two predictive models can be integrated, toxicity and permeability, using online application tools mentioned in material and methods. Potential antituberculosis compounds with higher activity can be identified from the Maybridge compound library by selecting those compounds that are closest to the active compounds (black spheres). We have used three computational models based on high throughput assays on *Mycobacterium tuberculosis* along with known and clinical trial drugs and the active set in addition we used a filter-based approach to filter out potential false positives/toxic molecules.

This straightforward approach is based on the fact that similarity in chemical properties is connected to the activity. The same principle can be employed for any database, simply by matching them with the compounds identified as most effective (black spheres in Figure 2). This approach will provide the chance to identify additional compounds with potential activities based on the similarity in their physicochemical properties. The latter is of particular importance because antituberculosis compounds must have a favorable pharmacokinetic profile, lower toxicity, and permeability. It is well known that the mycobacteria have special anatomical barriers that prevent simpler treatment. Such properties are ultimately related to the physicochemical properties of any compounds. Another potential application of this method is reversible screening by affording a direct match of a compound of interest to match similarity of its physicochemical properties with other libraries or datasets available, for example, natural products. The selected similar compounds from the reversible screening would have the advantages of sharing similar physicochemical properties. These compounds can be of any chemical classes and therefore bypass any limitation presented by the structural screening methods.

Researchers can benefit from this study by adopting their databases to ChemGPS-NP model to monitor their screening schemes at earlier stages by visualizing the distribution pattern and resemblance to the active set of compounds provided as Supplementary Materials. Furthermore, the list of highly active compounds can act as a reference set of compounds that can be matched with any database for screening enrichment and potential identification of antituberculosis compounds.

## 3. Material and Methods

### 3.1. Data Collection and Sources

#### 3.1.1. Screening Schemes

Publically available screening results from three different sources were used (collectively called screening sets). These are the GSK set (776 compounds) [18], the NIAID Set (3779 compounds) [19], and the GVK Bio Set (2880 compounds) [19]. All of these compounds showed antagonistic activities against tuberculosis, and their activities were averaged and categorized for the purpose of comparison.

#### 3.1.2. In-House Active Set

An in-house active set was constructed from the different antimycobacterial screening schemes along with the 46 clinically proven drugs publicly available [18,19]. The selection was based on higher activities against tuberculosis. The active set consists of 38, 132, and 289 compounds, corresponding to GSK, GV KBio, and NIAID respectively. The top 20 compounds were piperazine and naphthalene derivatives. The list of compounds is provided as Supplementary Materials. The compounds were sorted according to the activity and provided with three ChemGPS-NP scores along with permeability and toxicity filters in the Supplementary Materials. This active set was used in the visualization model of ChemGPS-NP.

#### 3.1.3. Database

MayBridge screening collection, with over 53,000 organic compounds of drug-like properties, was used in ChemGPS-NP visualization model. This collection was also used as an example of how to extract potential targets when compared to antimycobacterial screening schemes.

### 3.2. Compound Classification:

#### 3.2.1. Classification According to Activity 

The grouping was performed for most of the sets, dividing them into seven classes corresponding to AAA, AA, A, BBB, BB, B, and D, arranged logarithmically in order of the highest activity to the lowest activity. Classes BBB to D were regarded as ineffective and were eliminated from analysis. All activity classes were used in defining antituberculosis property space.

#### 3.2.2. Classification According to Predicted Permeability

Probability predictions for permeability for all compounds were provided by MycPermCheck [20]. This logistic regression modeling of the physicochemical properties sorts compounds into a list of small compounds ranked by their permeability into *Mycobacterium tuberculosis,* ranked from highest to lowest probability.

#### 3.2.3. Classification According to Toxicity Profile

The toxicities of compounds from different screening strategies were provided by the web application SmartsFilter (University of New Mexico; http://pasilla.health.unm.edu/tomcat/biocomp/smartsfilter). Each compound was marked pass or fail in the same regards.

### 3.3. Chemical Global Positioning System- Natural Product (ChemGPS-NP)

ChemGPS-NP [21] is a principal component analysis (PCA) [22] based global chemical positioning system tuned for exploration of biologically relevant chemical space. This is pursued through an eight-dimensional (8D) map with dimensions based on structure-derived physicochemical characteristics, for the ChemGPS-NP reference set of 1776 compounds. By calculating PCA prediction scores using a publicly available service (http://chemgps.bmc.uu.se), large sets of compounds can be mapped onto the 8D-map. Using this method, compounds from different screening sets were mapped together with reference sets with known antituberculosis mechanisms and activity degrees. Principal component and PCA score predictions were calculated, employing the software SIMCA-P+, with the training set ChemGPS-NP. Prior to PCA determination, all data were centered and scaled to unit variance.

#### Descriptors

Molecular and physicochemical properties were incorporated as chemically intuitive descriptors, described in detail by Larsson and co-authors [21]. The selected ChemGPS-NP descriptors were calculated for all sets of compounds using the software dragonX by Talete srl. (https://chm.kode-solutions.ne). Compounds were submitted on the basis of their structure information as simplified molecular input line entry specifications (SMILES; http://www.daylight.com/smiles/) and initially verified as described by Rosén and co-workers [23] to omit salts, hydration, stereochemical, and ionic information.

### 3.4. Data Analysis and Presentation:

#### 3.4.1. Calculation of Euclidean Distances

Euclidean distance estimates based on ChemGPS-NP principles were calculated according to an Excel formula that was used to relate a compound and core set compounds. The Euclidean distance was calculated between points P = (*p*_1_, *p*_2_, ..., *p_n_*) and Q = (*q*_1_, *q*_2_, ..., *q_n_*) in Euclidean *n*-space, as defined by

(1) $$d\left(p,q\right)=\sqrt{{\left(p1-q1\right)}^{2}+{\left(p2-q2\right)}^{2}+\ldots +{\left(pi-qi\right)}^{2}+\ldots +{\left(pn-qn\right)}^{2}}$$

For the purpose of placing progressively greater weight on objects that were farther apart, the square of the Euclidean distances were also calculated as

(2) $$d^{2}\left(p,q\right)={\left(p1-q1\right)}^{2}+{\left(p2-q2\right)}^{2}+\ldots +{\left(pi-qi\right)}^{2}+\ldots +{\left(pn-qn\right)}^{2}$$

#### 3.4.2. Calculation of the Tanimoto Coefficient

(3) $$T_{AB}=\frac{n_{AB}}{n_{A}+n_{B}-n_{AB}}$$

where *n_AB_* is the number of common bits set to 1 in both ligands *A* and *B*, and *n_A_* and *n_B_* are the number of bits set to 1 in ligands *A* and *B* individually.

## Supplementary Materials

The following are available online at https://www.mdpi.com/1420-3049/25/4/945/s1, Table S1: List of highly active compounds (Active set) along with thier 3D positions on the chemical property space in addition to the permiability and toxicity filters.

## Abbreviation

ChemGPS-NP, Chemical global positioning system–natural product; TB, tuberculosis; GSK, GlaxoSmithKline; GVKBio, GVK Biosciences; NIAID, National Institute for Allergy Infectious Diseases; PCA, principle component analysis; MDR, multidrug resistance; QSAR, quantitative structure–activity relations; HTS, high-throughput screening.
